# Modeling the Habitat Retreat of the Rediscovered Endemic Hawaiian Moth *Omiodes continuatalis* Wallengren (Lepidoptera: Crambidae)

**DOI:** 10.1371/journal.pone.0051885

**Published:** 2013-01-02

**Authors:** Adam E. Vorsino, Cynthia B. King, William P. Haines, Daniel Rubinoff

**Affiliations:** Department of Plant and Environmental Protection Sciences, University of Hawaii, Honolulu, Hawaii, United States of America; University of Lausanne, Switzerland

## Abstract

Survey data over the last 100 years indicate that populations of the endemic Hawaiian leafroller moth, *Omiodes continuatalis* (Wallengren) (Lepidoptera: Crambidae), have declined, and the species is extirpated from large portions of its original range. Declines have been attributed largely to the invasion of non-native parasitoid species into Hawaiian ecosystems. To quantify changes in *O. continuatalis* distribution, we applied the maximum entropy modeling approach using Maxent. The model referenced historical (1892–1967) and current (2004–2008) survey data, to create predictive habitat suitability maps which illustrate the probability of occurrence of *O. continuatalis* based on historical data as contrasted with recent survey results. Probability of occurrence is predicted based on the association of biotic (vegetation) and abiotic (proxy of precipitation, proxy of temperature, elevation) environmental factors with 141 recent and historic survey locations, 38 of which *O. continuatalis* were collected from. Models built from the historical and recent surveys suggest habitat suitable for *O. continuatalis* has changed significantly over time, decreasing both in quantity and quality. We reference these data to examine the potential effects of non-native parasitoids as a factor in changing habitat suitability and range contraction for *O. continuatalis*. Synthesis and applications: Our results suggest that the range of *O. continuatalis*, an endemic Hawaiian species of conservation concern, has shrunk as its environment has degraded. Although few range shifts have been previously demonstrated in insects, such contractions caused by pressure from introduced species may be important factors in insect extinctions.

## Introduction

Concerns over non-target effects of introduced biological control agents on native insects were most famously raised to the public conscience by conservationists in Hawaii [1]–[2]. The geographic isolation of the Hawaiian Islands has resulted in the evolution of a remarkable diversity of endemic organisms, and scientists expressed concern over the long-term threats to the persistence of these unique species [2]. Based on a review of biological control programs, and an assessment of the conservation status of endemic Hawaiian terrestrial arthropods, introduced biological control agents were postulated to be responsible for the extinction of at least 15 endemic Hawaiian Lepidoptera [1].

Hawaiian species in the moth genus *Omiodes* Guenée (Lepidoptera; Crambidae) have been at the forefront of heated debates concerning the safety and practicality of biological control on endemic organisms. In the early 1900s, two of the 23 Hawaiian species, the sugarcane leafroller (*O. accepta* Butler) and the coconut leafroller (*O. blackburni* Butler), became pests of economic significance on their respective crops [1], [3]. In response, between 1895 and 1960, several parasitoids were brought to the islands and released to suppress populations of the two endemic pests [3]. Subsequently, these introduced parasitoids were implicated in the disappearance and possible extinction of 14 non-target *Omiodes* species [1]. The release of these generalist parasitoids was conducted prior to the adoption of modern, more stringent and specific, biological control and risk assessment methodologies [4].

However, assertions of non-target impacts of the biological control agents were largely based on casual field observations, as opposed to research specifically designed to quantify non-target impacts [2], [5]–[6]. In response to this lack of data, retrospective studies were initiated over the last 20 years to assess the non-target effects of both accidentally and purposefully introduced parasitoids on current populations of several native Hawaiian insects [3], [7]–[9]. These studies confirmed that non-target attacks occur, but have only recently demonstrated that non-target parasitism is sufficient to cause population declines in native Hawaiian insects, though parasitoids may not be solely responsible for native insect extinctions [10], [11].

*Omiodes continuatalis* (Wallengren) is an endemic Hawaiian leafroller moth that feeds on both native and non-native grasses, and was formerly widely distributed across the Hawaiian Islands [12]. This moth was listed as extinct in the 1980s [1], and while it has since been rediscovered [13], surveys indicate that the species has declined across large portions of its original range [11], and like many endemic Hawaiian insects, it is of conservation concern. The moth was historically found on all of the main Hawaiian Islands, but now occurs only on the islands of Hawaii, Maui, Molokai, Lanai and Kahoolawe [11]. The reasons for the species’ extinction from relatively pristine reserves on the islands of Kauai and Oahu, and for its persistence in altered habitat on other islands, remain unclear.

Understanding why native species may be eliminated from nature reserves yet persist in altered landscapes is of broad importance to the fields of ecology and conservation. Conservation management often focuses on maintaining natural features of reserves, primarily native vegetation, but such features may not be the limiting factors defining suitable habitat for species of conservation concern. The impacts of invasive parasitoids on host species can vary greatly depending on the environmental conditions of the habitats where they occur, and parasitoids cause non-uniform range reductions in some species of Lepidoptera due to interactions with the local environment [14]. Because *O. continuatalis* appears to have suffered such non-uniform range reductions, it is an ideal candidate for examining how changes in habitat suitability over time, including parasitoid invasions, affect the persistence of a species across landscapes. The objectives of this study were to explore the spatial and temporal nature of the decline of *O. continuatalis* by examining changes in the presence and distribution of high quality habitat, as inferred from the historical and current presence of the moths. We used a retrospective ecological niche modeling (ENM) approach to reveal patterns of habitat overlap and movement.

Here we apply the maximum entropy approach to illustrate and quantify the probability of occurrence of a declining moth, *O. continuatalis,* across the Hawaiian Islands, and explore how the probability of occurrence has changed over time. By using projections of future climatic conditions, we also predict how habitat suitability is likely to continue to change in the future. We explore the reasons behind changes in habitat suitability, specifically referencing the distribution and impacts of introduced parasitoids.

## Materials and Methods

### Collections

#### Current survey data

**Figure 1:**
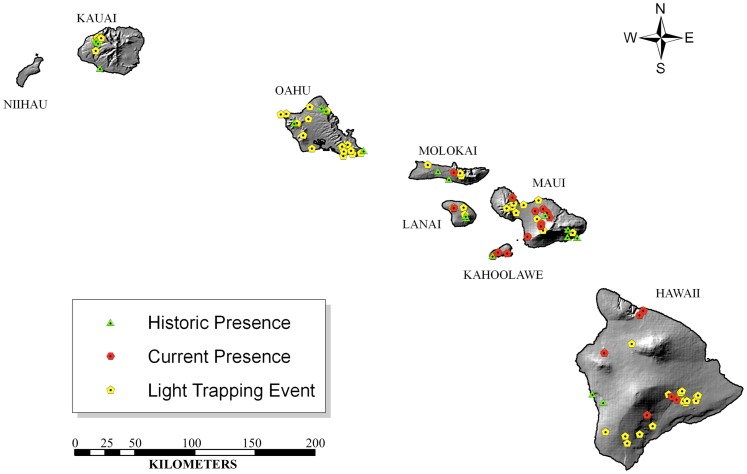
Map of historic and current *O. continuatalis* presence localities collected from around the Hawaiian Islands, as well as light trap collection localities where *O. continuatalis* were not observed during current surveys. These points were used to develop the Ecological Niche Models for each time period.

Current distribution data for *O. continuatalis* were obtained from field surveys at 111 locations, for 186 cumulative trap nights, across the Hawaiian Islands between 2004 and 2010 (Fig. 1). Locations for field surveys were selected based on the historical presence of *Omiodes* species, and/or the presence of *O. continuatalis* host plants. Ultraviolet (UV) lights were used either with bucket traps or on suspended white sheets to survey for *O. continuatalis* adults, with at least one of the two trap types set at each field site. These methods are effective for collecting *O. continuatalis*, which are strongly attracted to UV lights [11]. Moths observed but not necessarily collected during visual surveys were also included in these data. Specimens from current surveys were deposited at the University of Hawaii Insect Museum (UHIM). Only data points representing the localities where *O. continuatalis* were found (n = 19) (Fig. 1) were used in these analyses, because the modeling methods used rely on presence-only data (see below). Geographic coordinates were obtained for each of the localities using a Garmin eTrex Vista HCx Global Positioning System (GPS) unit, and by geocoding the localities using ArcGIS 10 [15] and Google Earth. All locality and environmental data were visualized in ArcGIS 10.

### Historical Survey Data

Historical *O. continuatalis* distribution data were obtained from the labels of *O. continuatalis* specimens deposited in the Bishop Museum insect collection (BPBM, Honolulu, HI). The Bishop Museum is the only significant source of historical locality data for *O. continuatalis*. Locality data (place name and elevation) for all *O. continuatalis* specimens collected during the period 1892–1967, were downloaded from the National Biological Information Infrastructure website (http://www.nbii.gov). Collections of *Omiodes continuatalis* occurred continuously over this 75-year period, by many different collectors who primarily used light traps, but apparently ceased in 1967 and were not resumed until our current surveys. Specimen collection locations were reviewed and geocoded using ArcMAP or Google Earth to obtain geographic coordinates. Specimens without adequate information to geo-reference localities within approximately 1 km were not used in the analyses. Where multiple specimens were available for the same locality, the locality data were geocoded only once. Out of 40 individual historical collections with adequately precise locality information, we derived point data for 19 localities across seven islands (Fig. 1).

### Habitat Suitability Analysis

#### Selection of key habitat variables

We selected and developed four environmental variables (elevation, vegetation, and proxies for precipitation, and temperature) that would maximize variance explained per site while also reducing over-parameterization and auto-correlation (Table 1). We chose a broad set of environmental attributes that varied on a relatively coarse spatial scale (30 arc seconds, or ∼ 1 km) because we had a small number of localities from which to define the ENM, and these were all based on light trap collections of adult moths (a coarser indicator of distribution than larval surveys). By incorporating only this subset of descriptive variables we created a liberal prediction of distribution compared to what might be predicted based on larval distribution data, which are unavailable for *O. continuatalis* due to their cryptic nature [16].

**Table 1:** An overview of the environmental variables used to define the ENM for *O. continuatalis.*

	Description	Source	Ecological Relevance	Variable type	Citation
**Elevation**	DEM: 10 meter digital elevation model of the Hawaiian Islands	http://ccma.nos.noaa.gov/products/biogeography/hawaii_cd_07/welcome.htm	Elevation is directly correlated in the distribution of many native and non-native insects and is definitive of microhabitat use [1], [70]–[73]	Continuous/Abiotic	[74]
**Vegetation Map**	Hawaii USGS 30 meter LANDFIRE Analysis Vegetation map	http://landfire.cr.usgs.gov/viewer/	Existing Vegetation Type analysis conducted for the Hawaiian Islands describing vegetation ecotypes based on canopy height and cover. It can used as a relatively large scale predictor of biotic trends [75]–[76].	Categorical/Biotic	[76]
**Current PCAtemp**	The first Eigenvector of a Principal Component Analysis of current (1950–2000) temperature	http://worldclim.org	Descriptive of the variance in temperature as described by the first eigenvector of Bioclimatic variables 1–12. Using this descriptor as a variable reduces multi-colinearity (redundancy).	Continuous/Abiotic	[17]
**Current PCAprecip**	The first Eigenvector of a Principal Component Analysis of current (1950–2000) precipitation	http://worldclim.org	Descriptive of the variance in precipitation as described by the first eigenvector of Bioclimatic variables 13–19. Using this descriptor as a variable reduces multi-colinearity (redundancy).	Continuous/Abiotic	[17]
**Future PCAtemp**	The first Eigenvector of a Principal Component Analysis of future (2050) temperature inferred from the GFLD 2.1 climate model A2a emission scenario.	http://www.ccafs *-climate.org/*	Used the same Bioclimatic variables as the current PCAtemp. A model evaluation conducted by Irving *et al.,* [18] indicated that the GFDL 2.1 climate model performed adequately for Pacific region projections.	Continuous/Abiotic	[77]
**Future PCAprecip**	The first Eigenvector of a Principal Component Analysis of future (2050) temperature inferred from the GFLD 2.1 climate model for the A2a emission scenario.	http://www.ccafs-climate.org/	Used the same Bioclimatic variables as the current PCAprecip. A model evaluation conducted by Irving *et al.,* [18] indicated that the GFDL 2.1 climate model performed adequately for Pacific region projections.	Continuous/Abiotic	[77]

The abiotic variables PCAPrcp and PCATemp are the first eigenvectors (EV's) of a Principal Component Analysis (PCA) conducted on a subset of the 19 current and future 30 arc second bioclimatic variables (www.worldclim.org and www.ccafs-climate.org) in the Spatial Analyst extension for ArcGIS 10. The worldclim dataset was developed from data collected between 1950 and 2000 [17], and was used to develop the ENM describing the contemporary and historic distributions of *O. continuatalis*.

Two PCAs were conducted such that the variance described by temperature (PCATemp = Bioclimatic variables 1–11) and precipitation (PCAPrcp = Bioclimatic variables 12–19) were explained by two separate analyses. Table 1 shows the manner in which the bioclimatic variables were parsed. The first EV of the precipitation PCA explained 99% of the variance in both the current and future datasets. For temperature, the first EV explained only 60.8% and 74.1%, while the second EV explained 39.1% and 25.8% of the variance in current and future climate datasets, respectively. Supporting information Tables S1A-D show the EVs from PCAs, and the proportional and cumulative variance explained by each. The future bioclimatic variables were derived from the 2050 GFDL 2.1 climate change model under the A2a emission scenario, which was described by Irving *et al.* [18] as appropriate for predicting the effects of climate change in the Pacific Islands.

Correlations between all pairs of environmental and climate variables (current and projected) were assessed using Pearson correlation analyses in ENMtools (vers. 2.1). Variables were selected for inclusion in analyses only if they were less than 35% correlated (positively or negatively) to another variable within the same time period (current or future) This is a conservative approach towards autocorrelation (i.e. multi-colinearity) considering other studies have used a selection threshold of less than 75% correlation [19]. The second EV's of both current and future temperature PCA's (i.e. PCAtemp 2) were removed due to their extensive overlap with elevation. Our final analysis consisted of three abiotic variables (elevation, PCAtemp, PCAprecip) and a single biotic variable (vegetation). As recommended by Phillips *et al.* [20], we used a categorical variable describing vegetation type to better define the distribution of *O. continuatalis*.

#### Analyses

In both agricultural and conservation management, ENM's have been used to predict the suitable habitat of an organism in either its ancestral region or novel areas of possible invasion [21]–[27]. Using the ENM approach, ecologists can infer inter- and intra-species fundamental niches [19], [28]–[29] prevalence [30]–[32], and niche overlap [33]. Though there are a large number of other modeling approaches capable of defining these habitat characteristics, the presence-only maximum entropy approach implemented in the program Maxent is one of the most widely used and accurate techniques [28], [34]–[38]. Using species presences and environmental variables (elevation, soil, temperature, landcover etc.). Maxent has been used to accurately estimate the ENM under maximum entropy from as few as five occurrence records [20]–[22], [29], [38].

As recommended by Pearson *et al.* [22], a presence only modeling technique was used, in part to account for difficulty of detecting *O. continuatalis.* Given the mobile nature of adult moths, the failure to collect *O. continuatalis* during a particular trapping event can imply false absence [39]–[42], making the presence-only modeling technique implemented in Maxent vers. 3.3.3 [20], [38], a more appropriate methodology for our data set [22], [43]. Using the presence only methodology implemented in Maxent we were able to compare historic, current, and future predictions (i.e. time series analysis).

We used the maximum entropy ENM technique to analyze historic and current data to describe the historic, current, and future distribution of *O. continuatalis*. The maximum entropy “machine learning” methodology models the distribution of environmental variables as extracted from occurrence localities over geographic space. The approach then compares this distribution to a null distribution of those environmental variables over the same geographic space using a set of background points, referred to as pseudo-absences. Using this modeling approach, model significance and validation estimates can be approximated. This methodological framework is discussed in more detail by Phillips *et al.* [20], Phillips & Dudík [38], Franklin [44], and Elith *et al.* [45].

#### Modeling parameters

Within Maxent the maximum number of background points was set to 10,000 with a regularization multiplier of one. Bootstrap analysis was conducted over 500 replicates and the output format was set to the logistic option to better visualize the data. We used the threshold rule of *equal training sensitivity and specificity* to model each distribution throughout Hawaii [46], [47]. *Equal training sensitivity and specificity* refers to the choice of a model that has an equal probability of being sensitive (predicting true presences) as it does of being specific (predicting true absences) [32], [46], [48]. For all other variables the recommended default settings were used, as described in Phillips *et al.* [20], [38]. Because we had acquired presence and absence data for the current distribution of *O. continuatalis,* a separate ENM analysis was conducted (not reported here) using the current data, where the default prevalence of 0.5 was changed to 0.22 (the number of presence points/total number of points). We do not report this assessment because it was found to overlap with the current ENM defining habitat under the default (0.5) prevalence. As such, we only report the model developed under the default prevalence to allow for direct comparisons among time series. The Habitat Suitability Index (HSI) per pixel was calculated in Maxent, where a value of 0 represented unsuitable habitat and a value of 1 represented completely suitable habitat. For ease of interpretation, each map was projected in ArcGIS on an HSI scale from 0 to 0.86, because 0.86 was the largest HSI reported in any of the analyses.

#### Model validation

The presence-only area under the curve (p-AUC) analysis describing the sensitivity and specificity of each ENM prediction was calculated from a random test percentage of 20% of the data. Further validation of the model was conducted following the protocols of Pearson *et al.* [22] in the program pValueCompute vers. 1 [22]. This Jackknife validation test describes the probability of successfully predicting a randomly selected occurrence point (q). The test generates a p-value describing the analyses significance when compared to a random distribution [22].

### Model Comparison

We used the niche overlap tool in ENMtools vers 1.3 [33] to calculate pairwise niche overlap between developed models. The niche overlap tool calculates two similarity metrics, the *D* and *I* statistics [49], both of which range from 0 to 1. Here we report the *I* statistic, as it is more appropriate for presence-only ENM analyses [49]. A relative rank test and niche breadth analysis were also conducted using ENMtools. The relative rank test is correlated with the *I* metric, but is a measure of habitat pairing over all the possible habitat patches [33]. This test outputs a value between 0 (no agreement) and 1 (complete agreement) [50].

The *I* and relative rank metrics were further analyzed with the niche identity test in ENMtools. The identity test assesses whether the habitat suitability scores defined in two different ENM's are ecologically significantly different. In this test, the actual niche overlap metric (i.e. similarity metric) is compared to a one-tailed normalized null distribution to assess significance of the analysis. The identity test used here compares the niche overlap or relative rank of either the current or historical distribution of *O. continuatalis* and that of a model developed from a random selection of both sets of occurrence localities [33], [49]. This identity test was replicated 100 times with 1000 Markov Chain Monte Carlo (MCMC) iterations, as recommended by Warren *et al.* [49].

We also conducted a niche breadth analysis using the Levin's niche breadth (B) analysis technique [51]. This metric describes the distribution of suitable habitat over the area tested. The Niche Breadth analysis is here used to define how the relationship of *O. continuatalis* to the environmental variables is changing over time. Like the relative rank test, niche breadth test values range from 0 (where only a single grid cell is considered suitable) to 1 (where all the grid cells are suitable) [33], [50].

The area (km^2^) of suitable habitat per island was then defined to better understand how the habitat is distributed throughout the islands, and how the habitat will change with time on each island. To quantify suitable habitat we first must define a threshold of habitat suitability; here suitable habitat was defined as all pixels with an HSI ≥0.5. This was not the threshold used in the Maxent model for validation estimates, rather we decided to use this cutoff for ease of interpretation. Area of suitable habitat was then estimated for each island in ArcGIS.

## Results

### ENM's and their Validation

**Figure 2:**
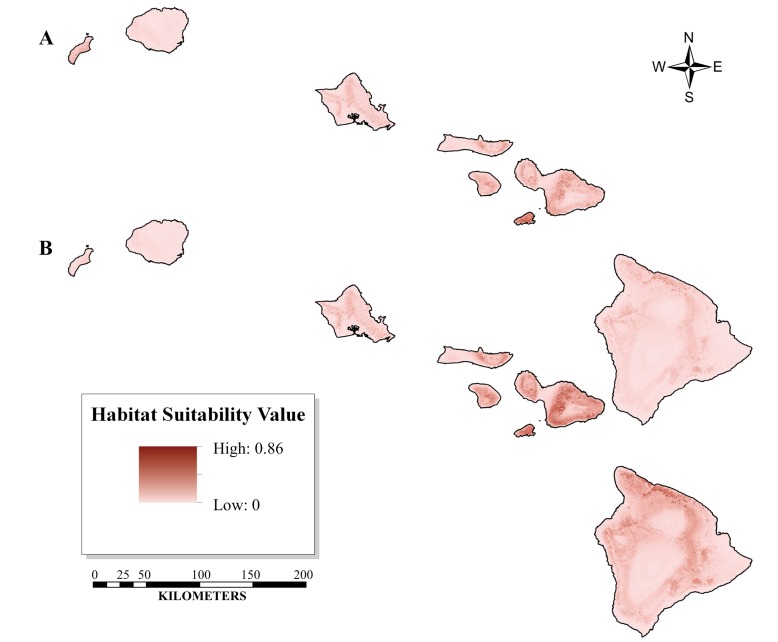
The Ecological Niche Models (ENM) defined in Maxent showing the distribution of *O. continuatialis* as collected from 2004 to 2010 and projected onto a set of biotic and abiotic variables. The figure shows the current ENM (A) along with that of the ENM projected into 2050 (B). The 2050 GFDL 2.1 climate change model was used to derive projected future climate variables. As compared to the current distribution (A) the 2050 projected distribution (B) shows an expansion of suitable habitat area (see Table 4).

**Figure 3:**
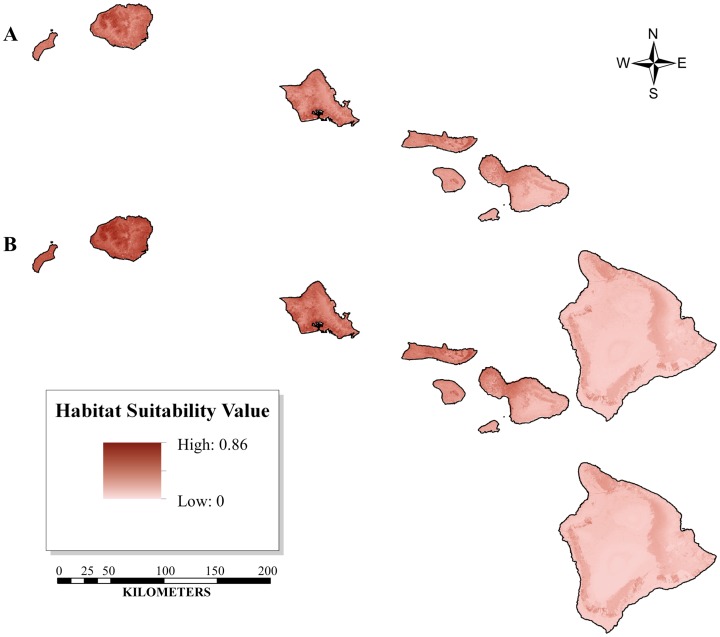
The Ecological Niche Models (ENM) defined in Maxent showing the distribution of *O. continuatialis* as collected from 1892 to 1967 and projected onto a set of biotic and abiotic variables. The figure shows the historically defined ENM (A) along with that of the ENM projected 2050 (B). The 2050 GFDL 2.1 climate change model was used to derive projected future climate variables. As compared to the historic distribution (A) the 2050 projected distribution of historic data (B) shows an expansion of suitable habitat area (see Table 4). Although the trend of habitat expansion is similar to the contemporary (current and projected) models, the overall model prediction differs significantly (Table 2).

Figures 2A–B and 3A–B show the ENMs developed for the historic, current, and future *O. continuatalis* distributions, each of which represents the mean of 500 spatial data models. The test p-AUC values ± the standard deviation averaged over all 500 model replicates are 0.861±0.118 for the current ENM and 0.804±0.124 for the historic ENM. Values of p-AUC greater than 0.8, such as those in this study, are indicative of highly specific and predictive models [28], [52]. The Jackknife validation test, an independent validation assessment for small sample sizes of each predicted distribution, was conducted in pValuecompute for each collection period (i.e. historic and current). This analysis is indicative of significantly predictive models when compared to a random distribution (p<0.0001 for both historic and current distributions). The Jackknife validation test predicted 78% of the historic and 69% of the current presence points used in the analysis.

### ENM Comparisons and Overlap

Table 2 shows the niche comparison metrics [49], as compared between current and historic collections under current and future climate scenarios. The greatest overlap was between projections of future distributions and the ENMs for the collection periods on which the future projections were based. The least overlap was between the 2050 projection based on current collection data and the ENM for the historic distribution. Interestingly, the identity test did not show ecologically significant differences between the current and historic distributions as defined for the current climate, yet there was a significant difference estimated when these same comparisons were conducted on the future climate projections (Table 2). The other comparison statistic used, the relative rank estimate, also showed high overlap between the current ENM and its future projection, and between the historic ENM and its future projection. This test showed relatively low overlap between current and historic ENMs. This would indicate that the current and historic habitat patches are not occupied in a similar manner. Both current and future climate projections for the current and historic data were found to be ecologically significantly different using the relative rank identity test (Table 2).

**Table 2:** Niche comparison metrics as calculated in ENMtools.

	Range Overlap		
	Contemporary Projection	Historic Analysis	Historic Projection
Contemporary Analysis	0.6228	0.0000	0.0071
Contemporary Projection	–	0.1249	0.1498
Historic Analysis	–	–	0.9978
	Relative Rank		
	Contemporary Projection	Historic Analysis	Historic Projection
Contemporary Analysis	0.8855	0.4896*	0.4881
Contemporary Projection	–	0.4252	0.4257*
Historic Analysis	–	–	0.9674
	Niche Overlap (*I*)		
	Contemporary Projection	Historic Analysis	Historic Projection
**Contemporary Analysis**	0.9840	0.8252 (n.s.)	0.8316
**Contemporary Projection**	–	0.7990	0.8068*
**Historic Analysis**	–	–	0.9992

Here the range overlap (difference in ranges between time periods), relative rank overlap (difference in habitat pairing between time periods), and niche overlap (*I*) (pairwise niche overlap between time points) metrics are shown. The results (significance or non-significance) of the identity test are also given for each applicable comparison (Relative Rank and Niche Overlap). Due to the nature of the identity test we could only compare point distributions under either the current or the future climate model, as such only two comparisons per model were conducted. Significance of the identity test (p<0.05) is indicated by “*”, whereas non-significance (p>0.05) is indicated by “n.s.”. The analysis indicates highly divergent Historic and Contemporary (current and projected) distributions of *O. continuatalis*.

The niche breadth analysis in Table 3 gives a good indication of habitat suitability/specificity over all the areas tested for each model. Interestingly, higher levels of suitability were defined by the future projections than the ENMs for current and historic distributions. This predicts an expansion of suitable habitat under projected future climate regimes, assuming all other variables remain the same.

**Table 3:** The Levin's Niche breadth analysis outputs a scale of specialization between 0 and 1, where “0” is a specialist and “1” is a generalist.

	Niche Breadth
	B1 (inverse concentration)
**Contemporary Analysis**	0.5067
**Contemporary Projection**	0.5444
**Historic Analysis**	0.6149
**Historic Projection**	0.6451

Here, niche breadth is used as a measure of the association to the environmental variables over time. Interestingly, the contemporary current and projected distribution of *O. continuatalis* shows lower niche specificity than does the historic distribution.

Yet a severe reduction in suitable habitat area is observed from historic to current time periods, suggesting that the range reduction from historical to current time periods is independent of climate change, perhaps due to changes in habitat or biotic interactions. As shown in Table 4, which compares suitable habitat by island, the total percent reduction in suitable habitat between historic and current distributions modeled under current climate conditions is 98.2%, and this difference remains substantial (90.2%) when modeled under future climate conditions. The differential between the historic and current models (current and future projections) corresponds to a total reduction of suitable habitat across all islands of −933 km^2^ and −1637 km^2^, respectively. Although a total reduction in habitat occurs for the summed area over all islands, some specific islands (i.e. Kahoolawe, Maui, Hawaii) experience an expansion of habitat area from historic to current periods modeled using current or future climate scenarios (Table 4).

**Table 4:** The area of suitable habitat (locations with an HSI>50%) per island per ENM in km^2^, the difference in these scenarios as compared between current and historic distributions, and their future (2050) projections.

	Area of Suitable Habitat (>50% HSI) per Analysis (km^2^)				Current Climate Models		2050 Future Climate Models	
Island	Historic ENM	Historic Projection	Contemp. ENM	Contemp.Projection	Δ Habitat Area (Contemp.-Historic)	% Δ Area	Δ Projected Habitat Area (Contemp.-Historic)	% Δ Area
**Niihau**	4.69	173.39	0.04	0	−4.65	−99.14	−173.39	−100
**Kauai**	575.47	1036.34	0	0	−575.47	−100	−1036.34	−100
**Oahu**	224.54	416.76	0	0	−224.54	−100	−416.76	−100
**Molokai**	55.58	82.28	0	0	−55.58	−100	−82.28	−100
**Lanai**	15.61	17.31	0.07	10.4	−15.54	−99.55	−6.91	−39.92
**Kahoolawe**	0	0	5.62	1.3	+5.62	+>100	+1.3	+>100
**Maui**	68.33	83.65	11.29	106.56	−57.04	−83.47	+22.91	+27.39
**Hawaii**	6.06	5.608	0.01	59.98	−6.05	−99.83	+54.372	+969.5
**Total**	950.28	1815.34	17.03	178.24	−933.25	−98.21	−1637.098	−90.18

The change (Δ) in habitat area is defined here by both percent change (%Δ Area) and the differences in the modeled ENMs. Totals are also defined to describe each distribution/projection in relation to the total area involved (all of the islands). The analysis indicates a large reduction in suitable habitat, though future projections of both current and historic *O. continuatalis* distributions indicate a habitat expansion.

## Discussion

The habitat utilized by a species is directly and indirectly influenced by abiotic environmental conditions. First, each species has a range of tolerance for abiotic environmental conditions, such as temperature and moisture, and a particular combination of these conditions is usually optimal for growth and reproduction. Second, adequate food resources, and the quality and abundance of these resources, whether they consist of detritus, specific host plants, or prey, are influenced by abiotic conditions. Finally, the range of a species is affected by pressures exerted by competitors, parasites, predators, and diseases, all of which are related to environmental conditions. A range shift in a species can therefore be driven by either a change in environmental conditions themselves (e.g. global warming or drought), or a change in any one of several indirect pressures that interact with environmental conditions (*e.g.*, the addition of a novel predator or competitor). Our objective in conducting the habitat suitability analyses was to quantify how the distribution, extent and quality of habitat for *O. continuatalis* has changed over time, and to explore possible explanations for why such changes may have occurred in light of the above mechanisms.

The predictive habitat distribution maps suggest that the current optimal habitat for *O. continuatalis* is significantly reduced compared to its historical range (Table 4). Additionally, these analyses suggest that localities where *O. continuatalis* is currently found are peripheral with respect to habitat considered optimal based on historical data (Figures 2 & 3). The average elevation ± standard deviation for historic optimal habitat, defined here as habitat with an HSI >0.5, is 665 (±396) m with a maximum elevation of 1,813 m. This contrasts with the contemporary average elevation, which is 541 (±207) m, with a maximum elevation of 1,167 m. The comparison indicates, for elevation at least, that the new optimal habitat is a contraction of the historic distribution.

We do not suspect that changes in food resources through habitat alteration are responsible for the shift in optimal range for *O. continuatalis*, although human land use impacts dominant vegetation and habitat structure, and can reduce or eliminate available resources, thereby reducing the persistence of populations [53]. For Lepidoptera restricted to feeding on rare or patchy resources, habitat destruction and alteration often translates into a decline or loss of larval host plants and adult nectar sources [54]–[56]. However, *O. continuatalis* is polyphagous on grasses, and readily utilizes several widespread non-native grass species, including hilo grass (*Paspalum conjugatum* Bergius) and kikuyu grass (*Pennisetum clandestinum* Chiov.) [12], which occur abundantly in many disturbed habitats, and are often the dominant groundcover of roadsides and pastures. Therefore, host plant distribution is not likely to be a direct limiting factor, although feeding on non-native host plants may influence the likelihood that non-native parasitoids will locate larvae. This hypothesis is supported by current survey data, which record *O. continuatalis* in habitats ranging from relatively intact native forest (Makawao Forest Reserve, Maui; Keamoku Flow, Hawaii Island) to highly disturbed agricultural areas (Haliimaile and Kula, Maui; Honokaa, Hawaii Island) and pasture (Haleakala Ranch, Maui) where native grasses do not occur. These data demonstrate that local extirpations of *O. continuatalis* populations do not correlate with habitat alteration through changes in land use.

Given that much of the optimal habitat contraction has been from lower to higher elevations, it is tempting to invoke climate change as a factor pushing *O. continuatalis* to higher elevations. In general, insect populations are expected to migrate to higher elevations and latitudes as global temperatures increase [57], and a warming trend has been documented in Hawaii [58], as well as changes in precipitation [59]. Although we would certainly expect changes in climate to influence habitat suitability, perhaps via interactions with parasitoids, the effect of climate change on suitable habitat between historical and current time periods is unclear. The historic and current ENM's were both inferred using the Worldclim dataset developed from data collected between 1950 and 2000 [17], which encompasses one of the most dynamic contemporary climatic periods ever recorded due to anthropogenic climate change [58], [60]. Although the shift in suitable habitat is consistent with what we might expect due to climate change, the projected 2050 ENMs using both current and historical collection data show an expansion of suitable habitat rather than a contraction, suggesting that the decrease in suitable habitat observed thus far is not simply a product of long-term climate trends.

If direct changes in abiotic factors are not responsible for the shift in habitat suitability, ecological interactions are another possible explanation. Introduced parasitoids and predators such as ants and vespid wasps are often assumed to be responsible for declines in Hawaiian Lepidoptera [1], [2]. Direct field observations confirm that *O. continuatalis* larvae are attacked by at least two ant species, *Pheidole megacephala* (Fabricius) and *Anoplolepis gracilipes* (Fr. Smith) [61], and they are likely attacked by other ant species as well. There are no native social insects in Hawaii, thus native insects are not evolutionarily equipped to defend against ant predation [62], [63]. There are at least 57 introduced ant species in Hawaii [64], and the highest diversity and density of ant species occurs at elevations below 900 m [65]. Given the extent of historical optimal habitat for *O. continuatalis*, ants have undoubtedly impacted lower elevation *O. continuatalis* populations. However the broader pattern of declines and extirpations of *O. continuatalis* is inconsistent with ant invasions across the Hawaiian Islands. *Omiodes continuatalis* has disappeared from apparently suitable habitats that currently have no ants, few ant species, or low densities of ants. For example, Kokee State Park on Kauai has experienced little invasion and establishment by ants [65], yet the results of extensive field surveys in the area indicate that *O. continuatalis* is no longer present, despite having been collected there until 1937. In contrast, field sites that have yielded the greatest number of *O. continuatalis* adults are infested with ants, including the voracious predator *P. megacephala* [61]. While the latter example shows only that *O. continuatalis* populations are able to withstand the effects of predation by ants in some habitats, the former example confirms that factors other than ants and habitat destruction are adversely affecting *O. continuatalis* populations.

Interactions with parasitoid wasps are also likely to have affected the range of *O. continuatalis,* and these interactions may be mediated by abiotic factors. Research over the last 20 years has confirmed that introduced biological control agents and adventive parasitoids utilize a broad range of native Hawaiian insects [3], [7]–[10]. These non-native parasitoid species have invaded relatively intact native ecosystems [7], [66], where other invasive predators have not established. Both accidentally and purposefully introduced parasitoid species are widely distributed throughout the Hawaiian Islands [67]. At least 42 non-native parasitoid species known to utilize Lepidoptera larvae or pupae have been collected from field sites across the Hawaiian Islands, and no fewer than 24 of them have been found inhabiting high elevation native forest habitats [7], [11], [20], [66].

In previous studies, the authors estimated parasitism rates on *O. continuatalis* at sites on Oahu and Maui using controlled exposure trials [61], in which sentinel eggs and larvae were exposed to parasitism. At least five non-native parasitoid species were found to attack *O. continuatalis*, and the estimated risk of parasitism was calculated to be higher at the Oahu site (65.3%) than at multiple Maui field sites where estimated risk ranged from 4.9%–27.4% [61]. An extensive metadata analysis of biological control introductions suggests that parasitism rates above 36% can independently suppress host populations [68]. While data from Maui indicate a risk of parasitism below this level, the 65.3% risk of parasitism for *O. continuatalis* on Oahu greatly exceeds this threshold. These data are consistent with the results of our ENMs, given that suitable habitat on Oahu has been more severely reduced than suitable habitat on Maui (Table 4).

Mortality from non-native parasitoids, if it is related to the environmental variables examined in this study, could explain the pattern of island extinctions that have been observed in *O. continuatalis* [11]. Recent and intensive survey efforts on Kauai and Oahu failed to locate *O. continuatalis*, even in localities where the species was previously collected [11]. The habitat suitability analyses suggest that there is no longer suitable habitat (HSI ≥0.5) for *O. continuatalis* on Kauai or Oahu, demonstrating a match between our model, field data, and expert opinion. More rigorous comparisons of parasitoid communities and rates of attack on the different islands are necessary to establish whether parasitism is an important factor in determining geographic range.

One important application of ENMs for threatened species is as a predictive tool to inform survey efforts. The ENM based on recent collection records identified suitable habitat in areas that have not been recently sampled, suggesting areas where remnant populations might be found. For instance, although there was no habitat considered suitable (HSI ≥0.5) on the island of Oahu, where this species is apparently extirpated, the best available habitat was concentrated in the central basin of the island, an area that has not been extensively surveyed. Similarly, the island of Niihau, which is privately owned and inaccessible to biologists and the general public, showed some suitable habitat. On Molokai, this species has been recently collected only once, from a well-sampled reserve in the central part of the island, yet the ENM suggests similarly suitable habitat might occur in the inaccessible eastern part of the island, where collection effort has been limited. In contrast, the island of Kauai showed very low habitat suitability, and may not be an ideal island on which to focus future survey efforts. The current ENM could also be used to identify the most viable locations for reintroduction efforts for *O. continuatalis*. Analyses of mitochondrial genetic data from this species has shown that populations exhibit both high levels of genetic diversity and almost no evidence of island-based genetic isolation, making it an ideal candidate for reintroduction on islands where it has been extirpated (W. Haines, unpublished data). Using the current ENM to identify introduction sites may increase chances of success.

### Conclusion

In this study we identified a trend of shrinking and shifting distributions of *O. continuatalis*, and discuss this trend in the context of experimental data from previous studies indicating spatially heterogeneous rates of attack by invasive parasitoids. While the Maxent approach is a promising tool to quantify changes in distribution of declining species like *O. continuatalis*, this model does not provide a means to determine the exact cause of the population decline. However, the model does elucidate an important temporal phenomenon: what used to be the highest quality habitat for *O. continuatalis* is now less frequented by the species, and the moths are now largely found in what was, based on historical data, lower quality habitat. This paradigm has been shown in birds [69] and demonstrates that current refugia may not represent the most favorable combination of abiotic variables under natural conditions, since present distributions might be determined by novel ecological interactions, not abiotic factors such as climate.

The ENM approach can be very important in drawing attention to cryptic causes for the disappearance of species of concern from historically suitable parts of their range. This is an essential first step in effectively conserving and reintroducing threatened species. The ENMs produced by Maxent analyses can be used to target areas for future surveys, or to identify potential sites of reintroduction for example, on islands where *O. continuatalis* has been extirpated. Our findings may also influence broader programs targeting the conservation of native insects or other declining species for which detailed ecological data is lacking, or hard to collect, since the ENM methodology is applicable to most habitat assessment efforts.

## Supporting Information

Table S1 **A–D: The results of the Principal Component Analysis (PCA) of precipitation and temperature variables (i.e. Bioclim 12–19 and 1–11, respectively) for both contemporary (A & C) and future (B & D) projections.** The Eigenvalue of each principle component is indicated, along with the proportional and cumulative variance explained.
(DOCX)
