# Supplementary material for: Modeling the Habitat Retreat of the Rediscovered Endemic Hawaiian Moth Omiodes continuatalis Wallengren (Lepidoptera: Crambidae)
Source: PLoS One. 2013 Jan 2;8(1):e51885. doi: 10.1371/journal.pone.0051885 (PMC3534676; doi:10.1371/journal.pone.0051885)
Supplement: Table S1 — A–D: The results of the Principal Component Analysis (PCA) of precipitation and temperature variables (i.e. Bioclim 12–19 and 1–11, respectively) for both contemporary (A & C) and future (B & D) projections. The Eigenvalue of each principle component is indicated, along with the proportional and cumulative variance explained. (DOCX) [file pone.0051885.s001.docx]

| **Precipitation** |  | | | | | | | | | | | | |  |
| --- | --- | --- | --- | --- | --- | --- | --- | --- | --- | --- | --- | --- | --- | --- |
| 1. **Contemporary** | **PCA1** | **PCA2** | | **PCA3** | | **PCA4** | **PCA5** | | **PCA6** | | **PCA7** | **PCA8** | | **Sum** |
| Eigenvalue of Precipitation | 95956.730 | 599.546 | | 38.040 | | 25.609 | 7.867 | | 5.579 | | 3.754 | 0.000 | | 96637.125 |
| Proportional Variance Explained | 0.993 | 0.006 | | 0.000 | | 0.000 | 0.000 | | 0.000 | | 0.000 | 0.000 | | 1.000 |
| Cumulative Variance Explained | 0.993 | 0.999 | | 1.000 | | 1.000 | 1.000 | | 1.000 | | 1.000 | 1.000 | | - |
| 1. **Future** |  | | | | | | | | | | | | | |
| Eigenvalue of Precipitation | 94939.330 | 642.467 | | 150.737 | | 40.875 | 30.153 | | 5.940 | | 4.977 | 2.631 | | 95817.110 |
| Proportional Variance Explained | 0.991 | 0.007 | | 0.002 | | 0.000 | 0.000 | | 0.000 | | 0.000 | 0.000 | | 1.000 |
| Cumulative Variance Explained | 0.991 | 0.998 | | 0.999 | | 1.000 | 1.000 | | 1.000 | | 1.000 | 1.000 | | - |
| **Temperature** |  |  |  | |  |  |  |  | |  |  |  |  |  |
| 1. **Contemporary** | **PCA1** | **PCA2** | **PCA3** | | **PCA4** | **PCA5** | **PCA6** | **PCA7** | | **PCA8** | **PCA9** | **PCA10** | **PCA11** | **Sum** |
| Eigenvalue of Temperature | 1544.547 | 994.085 | 1.712 | | 1.031 | 0.735 | 0.136 | 0.035 | | 0.012 | 0.007 | 0.000 | -0.025 | 2542.275 |
| Proportional Variance Explained | 0.608 | 0.391 | 0.001 | | 0.000 | 0.000 | 0.000 | 0.000 | | 0.000 | 0.000 | 0.000 | 0.000 | 1.000 |
| Cumulative Variance Explained | 0.608 | 0.999 | 0.999 | | 1.000 | 1.000 | 1.000 | 1.000 | | 1.000 | 1.000 | 1.000 | 1.000 | - |
| 1. **Future** |  | | | | | | | | | | | | | |
| Eigenvalue of Temperature | 2833.852 | 984.913 | 2.447 | | 1.142 | 0.777 | 0.190 | 0.055 | | 0.012 | 0.007 | 0.004 | 0.000 | 3823.398 |
| Proportional Variance Explained | 0.741 | 0.258 | 0.001 | | 0.000 | 0.000 | 0.000 | 0.000 | | 0.000 | 0.000 | 0.000 | 0.000 | 1.000 |
| Cumulative Variance Explained | 0.741 | 0.999 | 0.999 | | 1.000 | 1.000 | 1.000 | 1.000 | | 1.000 | 1.000 | 1.000 | 1.000 | - |

**Table S1 A-D: The results of the Principal Component Analysis (PCA) of precipitation and temperature variables (i.e. Bioclim 12-19 and 1-11, respectively) for both contemporary (A & C) and future (B & D) projections. The Eigenvalue of each principle component is indicated, along with the proportional and cumulative variance explained.**
